# Retinoic Acid Can Exacerbate T Cell Intrinsic TLR2 Activation to Promote Tolerance

**DOI:** 10.1371/journal.pone.0118875

**Published:** 2015-03-31

**Authors:** Vivien Nguyen, Kandyce Pearson, Jee-Hyun Kim, Karishma Kamdar, R. William DePaolo

**Affiliations:** 1 Department of Pediatric Gastroenterology and Nutrition, Children’s Hospital of Los Angeles, Los Angeles, California, United States of America; 2 Department of Molecular Microbiology and Immunology, Keck School of Medicine and University of Southern California, Los Angeles, California, United States of America; Centre d'Immunologie de Marseille-Luminy, CNRS-Inserm, FRANCE

## Abstract

The contribution of vitamin A to immune health has been well established. However, recent evidence indicates that its active metabolite, retinoic acid (RA), has the ability to promote both tolerogenic and inflammatory responses. While the outcome of RA-mediated immunity is dependent upon the immunological status of the tissue, the contribution of specific innate signals influencing this response have yet to be delineated. Here, we found that treatment with RA can dampen inflammation during intestinal injury. Importantly, we report a novel and unexpected requirement for TLR2 in RA-mediated suppression. Our data demonstrate that RA treatment enhances TLR2-dependent IL-10 production from T cells and this, in turn, potentiates T regulatory cell (T_REG_) generation without the need for activation of antigen presenting cells. These data also suggest that combinatorial therapy using RA and TLR2 ligands may be advantageous in the design of therapies to treat autoimmune or inflammatory disease.

## Introduction

The general concept that Vitamin A (VA) contributes to immunity dates as far back as Hippocrates [1], and recent advances have demonstrated specific roles for VA in many different types of disease. For instance, VA deficiency (VAD) increases mortality during gastrointestinal, respiratory and HIV infections [2–5] which can be reversed by VA supplementation [6–7]. Despite these observations the role of VA is still not well understood in the context of intestinal inflammation even though more than 15% of children with inflammatory bowel disease (IBD) have low serum levels of VA at the time of diagnosis [8].

VA mediates its metabolic and immune effects via conversion to its active metabolite, RA, via retinaldehyde dehydrogenase (RALDH) enzymes [9–11]. In the last decade, many studies have provided insight into the nature of RA-mediated responses, especially its role in innate and adaptive immunity within the gut associated lymphoid tissues (GALT). Most notably, RA promotes T cell trafficking to the GALT via α4β7 and CCR9 expression [12–14] and contributes to the polarization of Foxp3^+^ T_REG_ by RALDH-expressing CD103^+^ GALT DC [15–20]. These effects are dependent on TGF-β mediated T cell expression of retinoic acid receptor (RAR) and repression of the IL-6R, respectively [21–23]. Corroborating these *in vitro* findings, the generation of induced T_REG_ (iT_REG_) in response to ingested antigens is abrogated in VAD mice [24].

iT_REG_ and IL-17-producing CD4 helper T cells (T_H_17) have a reciprocal relationship [19, 25, 26], leading one to expect an inhibitory effect of RA on T_H_17 differentiation and maintenance. A number of studies have shown that direct activation of RA on T cells can suppress T_H_17 differentiation through the inhibition of IL-6R and IL-23R [13,19,23]. However, antigen-presenting cells activated via MyD88-dependent innate signals and treated with RA have been shown to potentiate T_H_17 differentiation [27]. These data suggest that RA, in concert with microbial-driven signals, may help to promote T_H_17 cell differentiation further suggesting that RA may have a dual nature imparting it with the ability to both promote and inhibit iT_REG_ generation via the regulation T_H_17 cells [28].

Pathogens crossing the epithelial barrier during infection or exposure of the tissue to commensal bacteria during injury can provide the microbial signals needed to impact RA-mediated immunity. While tissue-derived homeostatic factors may promote the expression of RALDH in CD103^+^ DC in order to potentiate iT_REG_ cell numbers [29,30,31], inflammation and exposure to microbes may have an opposite effect. This has been observed in models of experimental colitis in which the expression of RALDH in CD103^+^ DC is reduced resulting in fewer iT_REG_ and worse inflammation [32]. In an IL-15-enriched microenvironment, RA was shown to increase the production of IL-12 and IL-23 by gut CD103^+^ DC, diminishing their capacity to promote iT_REG_ and restrain T_H_1 and T_H_17 responses to dietary gluten [33]. These data align with clinical reports linking pharmacological retinoid therapy to the development of IBD in a subset of patients and point to RA as a potential instigator of inflammation in the appropriate milieu [24,34].

TLR2 is a member of the Toll-like receptor (TLR) family of pattern recognition receptors, and detects tri- [35] and di-acylated [36] bacterial lipoproteins by forming heterodimers with TLR1 or TLR6, respectively. TLR2 signaling in splenic DC induces RALDH activity [30] and IL-10 [37], imparting them with gut-specific imprinting and iT_REG_-promoting functions. In contrast, others have demonstrated preservation of RALDH activity in MyD88-deficient DC [38] and promotion of T_H_17 cells [27] and RALDH [32] during microbial stimulation. Studies examining the relationship between TLR2 and RA have focused on the DC, despite reports that TLR2 is expressed on T_REG_ [39] and may influence T_REG_ expansion and function [40–42]. Here we show that exogenous RA can suppress inflammation during intestinal injury and that this ability is lost in a TLR2-deficient environment. Further, we show that RA potentiates TLR2-induced IL-10 production directly from T cells and promotes iT_REG_ differentiation. These findings further demonstrate the ability of RA to act as an adjuvant to promote signals from the local tissue microenvironment and suggest a potential benefit of combining TLR2 ligands and RA to suppress inflammatory disease and promote tolerance.

## Materials and Methods

### Mice

This study was performed in strict accordance and compliance with the recommendations in the Guide for the Care and Use of Laboratory Animals of the National Institutes of Health, the Animal Welfare Act and U.S. Government Principles for The Utilization and Care of Vertebrate Animals Used in Testing Research and Training. All studies were approved and in accordance with the Institutional Biosafety Committee (IBC) and the Institutional Care and Use Committee (IACUC) of the University of Southern California. Mice were housed in specific pathogen free (SPF) conditions.

C57Bl/6 (WT) mice were purchased from The Jackson Laboratory (Bar Harbor, ME) and TLR2KO mice on a B6 background were bred in-house. Male mice aged 6–8 weeks were used for all experiments and the numbers of mice used for each experimental group are listed in the figure legends.

### Induction of colitis

2.5% (weight/volume) dextran sodium sulfate (DSS; molecular weight 36,000–50,000; lot NM4241; MP Biomedicals; Santa Ana, CA) was added to drinking water for 7 days, followed by a 7-day period of recovery with untreated drinking water.

### Clinical and histological evaluation of colitis

Body weight, presence of occult or gross blood per rectum (scoring: 0, no blood; 1, occult blood positive; 2, visible blood in fecal pellet; 4, blood at anus) were determined daily. Colonic specimens were opened longitudinally and made into “Swiss-roll” preparations. Hematoxylin and eosin staining was performed on 5μm paraffin-embedded sections. Slides were viewed with a Leica DM 750 microscope with PLAN 10x/0.22 and PLAN 40x/0.65 objectives. Histologic severity was assessed using a scoring system developed at The Jackson Laboratory [42]. All slides were evaluated in a blinded manner by independently two gastroenterologist/pathologists.

### Oral tolerance induction

WT and TLR2KO mice were orally gavaged as previously described [33]. Fecal occult blood tests (Hemoccult Sensa II; Beckman Coulter; Brea, CA) were performed daily and upon conversion from negative to positive, 1 μM of RA dissolved in 100 μl of corn oil (Sigma-Aldrich; St. Louis, MO) was administered to each mouse via oral gavage with an 18 gauge round-tipped needle (Kent Scientific Corp; Torrington, CT) for the duration of the experiment. Controls received the same volume of DMSO diluted in 100 μl of vehicle (corn oil). In some experiments the mice received 100 μl of sterile PBS containing 100 μg of ovalbumin or PBS alone every other day for a total of five feedings. Mice were euthanized two days after the last feeding. In some experiments 1x10^5^ syngeneic ovalbumin-specific T cells from RAG-OT2-Tg mice were transferred to naïve TLR2KO mice prior to antigen feeding via retro-orbital injection [33].

### Protein extraction from tissues and cecal lysates

Total protein was extracted from whole colon specimens by flash freezing with liquid nitrogen and manually homogenizing with a mortar and pestle. For cecal lysates, total contents were placed in 500 μl sterile PBS and mixed with 500 μl 0.1 mm glass beads and mechanically homogenized for 3–4 minutes using a Bead Beater (BioSpec). After beads and debris settle to the bottom of the tube, the supernatant is removed. Protein concentrations of supernatants were determined via Bradford assay per manufacturer’s protocol (Bio-Rad; Hercules, CA) and homogenates were diluted to a concentration of 1 mg/mL with sterile PBS (Sigma-Aldrich).

### Quantitative real-time RT-PCR

*Q*uantitative real-time RT-PCR was performed using the iCycler iQ real-time PCR detection system (Bio-Rad Laboratories) and a SYBR green amplification kit (PE Biosystems). Each PCR reaction was performed and normalized using primers for GAPDH. *ΔΔCT* was calculated using *ΔC*T values of naive WT or naïve TLR2KO samples as the reference.

### In vitro assays

For CD4^+ ^T cell isolation, spleens were mechanically disrupted through a 70 μm cell strainer (BD Biosciences; San Jose, CA). CD4^+^ cells were isolated by positive immunoselection using CD4 (L3T4) microbeads (Miltenyi Biotec; Bergisch Gladbach, DE). Purified CD4^+ ^T cells were stained with anti-CD62L and anti-CD44 and sorted on a FacsAria II resulting in a 98% pure population of naïve T cells. The purified T cells were stimulated with 1 μg/mL plate-bound anti-CD3ε (eBioscience; San Diego, CA), 0.1 μg/mL TLR ligands, 10 μg/mL cecal lysate, and 2 ng/mL recombinant TGF-β (R&D Systems; Minneapolis, MN), in the presence or absence of 10 nM RA. In some experiments 50 μg/ml anti-IL-10Rα antibody (clone 1B1.3a) or isotype rat IgG1 (BD Biosciences) control was added to the wells.

For dendritic cell isolation, spleens were digested with 400 units/mL collagenase type IV (Sigma-Aldrich). Cells were filtered, re-suspended in 22.5% Optiprep (Sigma-Aldrich), overlaid with Hank’s Buffered Saline (HBS; Sigma-Aldrich) and centrifuged at 670*g* for 30 minutes. Dendritic cells were sorted from the interphase using magnetic CD11c Microbeads (Miltenyi Biotec; Bergisch Gladbach, DE) and stimulated with 10 μg/mL cecal lysate and 2 ng/mL TGF-β in the presence or absence of 10 nM RA.

### Antibodies and flow cytometry

The following conjugated antibodies were purchased from eBioscience: CD4 (GK1.5), CD45.1 (A20), CD45.2 (104), IFN-γ (XMG1.2), Foxp3 (FJK-16a), IL-10 (JES5-16E3) and isotype controls. A fixation and permeabilization kit was used for intranuclear detection of Foxp3 followed by an intracellular staining protocol for IL-10 (eBioscience). Flow cytometry analysis was performed with a FACS Canto (BD Biosciences) and analyzed using FlowJo software.

### Detection of cytokines by ELISA

Cell supernatants (50 μL per well) and tissue homogenates (50 μg per well) were evaluated for IL-12p40 (BD Biosciences), IL-10, (BD Biosciences), TNF-α (BD Biosciences), IFN-γ (BD Biosciences) and IL-17 (R&D).

### Statistical analysis

Data are expressed as means ± standard error of the mean (SEM) of 2 or more independent experiments. Differences between means are evaluated using 2-tailed, unpaired Student’s *t*-tests (GraphPad Prism version 6; San Diego, CA) where appropriate. *p* values of < 0.05 are considered significant.

## Results

### RA-mediated repair against epithelial injury is dependent on TLR2-signaling in vivo

**Fig 1:**
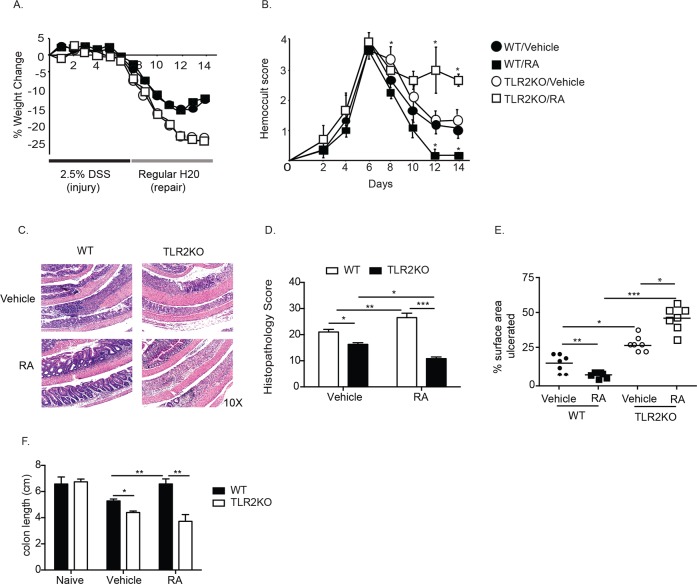
RA potentiates colonic injury and inflammation in the absence of TLR2 signaling. WT and TLR2KO mice were given 2.5% DSS for seven days to induce colonic damage and then placed on normal drinking water for seven days to allow for tissue repair. Upon first presence of fecal occult blood positivity for each, mice were supplemented with RA or vehicle control, which continued for the rest of the disease course. Weight loss (A) and fecal occult blood (B) (n = 10–14 mice per group) were monitored daily. (C) H&E staining of colonic sections of mice at day 14 (after 7 days of DSS followed by 7 days of water). (D) Histology scoring of H&E-stained colonic slides (n = 4 mice per group). (E) The percent of surface area containing ulcerated/denuded epithelium quantified using J Image software. (F) Colon lengths from mice at day 10 (n = 5–6 mice per group). *, *p* < 0.05, **, p < 0.01 using Students t-test.

Due to the prevalence of vitamin and micronutrient deficiencies in patients with IBD, enteral repletion is often initiated at the time of diagnosis [8]. Given the potential for RA to promote either regulatory [13,19,23] or inflammatory [24,33] immune responses depending on the status of the tissue microenvironment, we assessed the effects of RA supplementation during acute colitis and evaluated the role of TLR2 in modulating these effects. DSS was administered to WT and TLR2 deficient mice to induce epithelial injury for seven days followed by a repair phase in which the mice received regular drinking water. In order to mirror the clinical presentation and course of individuals with IBD, we delayed enteral supplementation of RA until overt signs of colitis (i.e. loose stool and fecal occult blood) were noted. In line with prior studies demonstrating that the absence of TLR2 signaling exacerbates chemically-induced and spontaneous colitis in mice, we found that TLR2KO mice lost more body weight than WT counterparts during induction of colitis as well as during recovery [43,44] (Fig 1A, closed and open circles). TLR2KO mice also had significantly worse fecal occult blood scores (Fig 1B, closed and open circles), and worse histopathology (Fig 1C) compared to WT mice, with larger areas of denuded, ulcerated epithelium (Fig 1D and 1E, white bars, closed and open circles) and shorter colon lengths (Fig 1F), indicative of inflammation. WT mice treated with RA showed significant improvement in fecal occult bleeding (Fig 1B, closed squares), histopathology (Fig 1C, bottom left), less epithelial damage (Fig 1D and 1E, black bar and closed squares) and significantly longer colons (Fig 1F). In contrast, RA supplementation had adverse effects in TLR2KO mice, which demonstrated severe rectal bleeding (Fig 1B, open squares); worse histopathology (Fig 1C and 1D, bottom right, black bars), and shorter colons (Fig 1F) compared to both WT mice receiving RA and TLR2KO mice receiving vehicle control. Together these data demonstrate that treatment with RA can protect against epithelial injury during acute colitis however, in the absence of TLR2, treatment with RA exacerbates disease.

### RA mediated suppression of colonic cytokine production in vivo is dependent on TLR2

**Fig 2:**
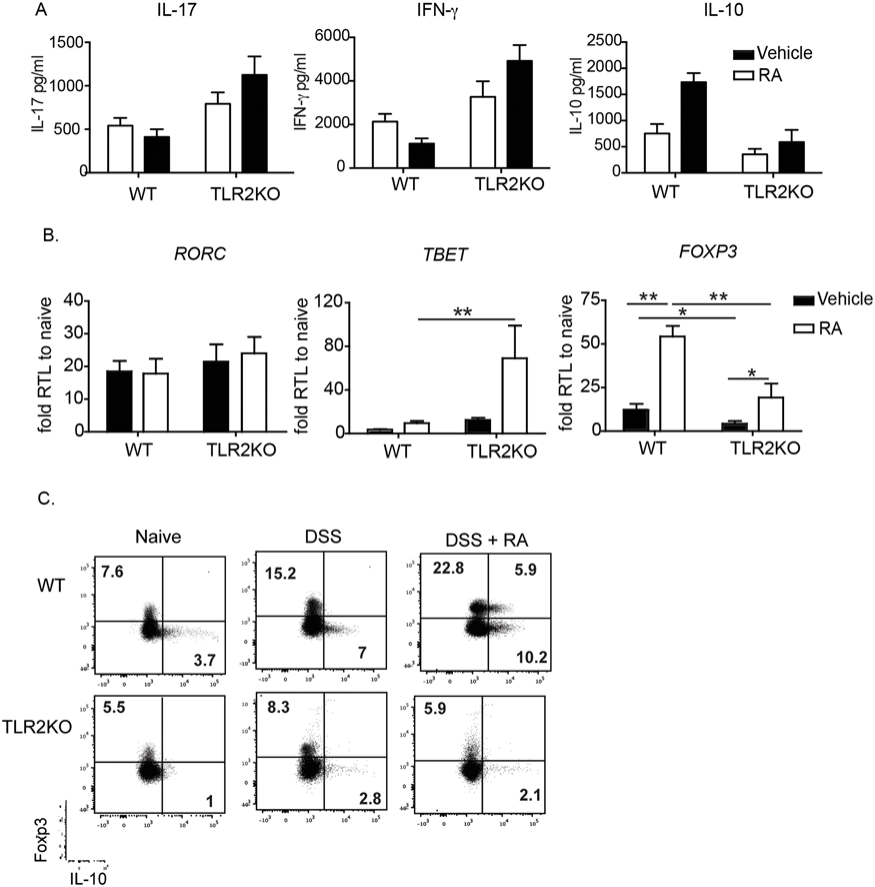
RA treatment suppresses IFN-γ during DSS in WT mice but enhances their secretion in TLR2KO mice. (A) Mucosal scrapings from mice receiving the DSS and water were harvested on day 10 and analyzed by ELISA for cytokine levels. (B) Quantitative RT-PCR for transcription factors associated with T helper subsets were also performed on colonic lamina propria samples taken at day 14. Data shown are the fold increase of Vehicle- and RA-treated mice compared to naive controls. Data are the mean ± SEM of 5–8 mice per group pooled from two independent experiments. (C) Expression of Foxp3 and IL-10 in colonic LP CD4^+^ T cells (n = 3 per group), one representative facs plot is shown. *, p < 0.05, **, p < 0.01 using Student’s t-test.

To determine if RA supplementation affects inflammatory cytokine production, we examined the levels of cytokines in the colonic mucosa at day 10. RA treatment suppressed IFN-γ (Fig 2A) and TNF-α (data not shown) production in WT mice and exacerbated their production in TLR2KO mice (Fig 2A). In contrast, RA treatment increased IL-10 production in the WT mice, an effect that was not observed in the TLR2KO mice (Fig 2A), corroborating earlier observations that TLR2 directly promotes IL-10 responses [48]. RA had no effect on IL-17 production in either condition (Fig 2A).

The colonic mucosa was analyzed for mRNA expression of T-helper associated transcription factors at day 14. Complimenting the cytokine data described above, RA treatment exacerbated the expression of *TBET* in TLR2KO but not WT mice. Furthermore, although RA increased *FOXP3* expression in both conditions, its expression was significantly lower in TLR2KO animals (Fig 2B). As seen with the cytokine analysis, RA treatment had no impact on the T_H_17-associated factor *RORC*. The increased IL-10 and *FOXP3* levels lead us to speculate that the protective effect of RA observed in WT mice may be due to the induction of anti-inflammatory responses via the induction of IL-10 and Foxp3^+^ T regulatory cells. Examination of colonic LP cells 10 days after initial DSS treatment demonstrated an increase in the frequency of CD4^+^ T cells expressing Foxp3^+^ and IL-10 in WT mice treated with RA, an effect that was not in TLR2KO mice (Fig 2C). Taken together, these data suggest that *in vivo* RA treatment affects both cytokine production and T cell polarization in a manner dependent upon TLR2 signaling.

### The suppressive effect of RA and TLR2 is not mediated by dendritic cells

**Fig 3:**
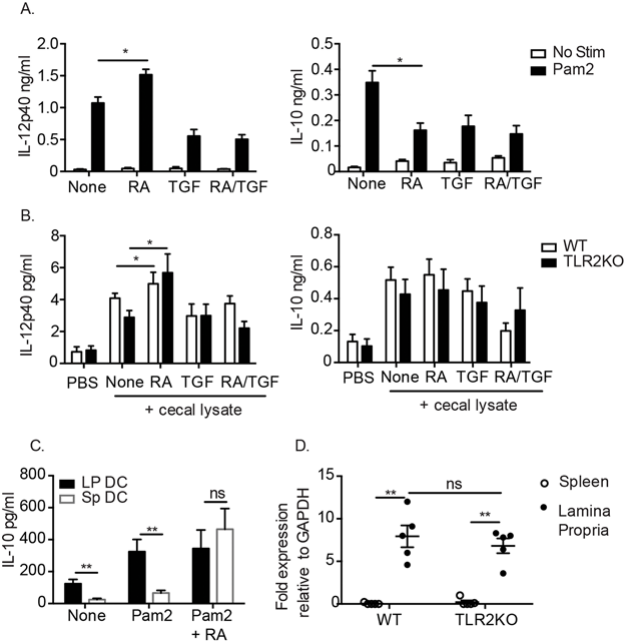
RA potentiates cytokine responses in both WT and TLR2KO DC. (A) WT splenic DC were cultured with 100 ng/ml of TLR2 ligand Pam2CysK4 and (B) WT and TLR2KO splenic DC were cultured with 10 μg/ml cecal lysate in the presence or absence of RA, TGF- β or RA/TGF- β. Supernatants were analyzed after 24 hours for cytokine production by ELISA. (C) Comparison of IL-10 IL- production from CD103^+^ LP DC and CD103^+^ SP DC. (D) Transcript levels of *aldh1a2* in spleen and LP of naïve WT and TLR2KO mice determined by quantitative PCR. For (A-C), data are the mean ± SEM of three independent experiments, for (D) 5 individual mice were analyzed. *, p < 0.05, **, p < 0.01 using Student’s t-test.

Both TLR2 and RA have been shown to have effects on DC as well as T cells [21,23,27,37]. In order to further dissect the role of RA and TLR2 in regulating cytokine production, splenic DC were purified from the spleens of WT and TLR2KO mice. To evaluate the specific effects mediated by RA and TLR2 on DC in a gut environment [33] TGF-β and the TLR2/6-agonist Pam2Cysk4 (Pam2) were added to cultures containing RA. RA treatment suppressed Pam2 mediated IL-10 production by WT DC, and enhanced IL-12p40 production (Fig 3A). RA treatment had no effect on the production of these cytokines by WT DC in the absence of Pam2, or in TLR2KO DC (data not shown).

To determine whether specific, endogenous signals within the microbiota or DC-modulators produced by the epithelium can alter the response to RA in a TLR2 dependent manner, we treated splenic DC from WT and TLR2KO mice with lysates of their own cecal contents. RA exacerbated the production of IL-12p40 by both WT and TLR2KO DC stimulated with cecal lysates, however there was no impact of RA on IL-10 in these conditions (Fig 3B). The intestinal microenvironment differs greatly from peripheral tissues such as the spleen, to determine whether the effects of TLR2 and RA would also occur in mucosal derived cells we sorted and stimulated CD103^+^ DC from the colonic LP and spleen as previously described [33]. CD103^+^ LP DC will produce RA *ex vivo* [18, 20] and the addition a TLR2 agonist produced levels of IL-10 equal to splenic CD103^+^ DC plus TLR2 agonist and RA (Fig 3C) suggesting that the ability of TLR2 to induce IL-10 is not tissue specific. Previous studies have also indicated that TLR2 signaling in splenic DC induces RALDH which converting vitamin A to RA [30]. We evaluated the transcript levels of *aldha1a2* in splenic and LP DC to determine whether TLR2KO mice had an intrinsic defect in RA levels accounting for the phenotype we observed. However, we found similar levels of *aldha1a2* regardless of TLR2 expression (Fig 3D). Overall, RA was unable to suppress *ex vivo* IL-12p40 production by WT splenic DC stimulated with either a TLR2-specific agonist or commensal lysate and had little effect on cytokine production by TLR2KO DC.

### RA enhances TLR2-induced IL-10 production from CD4^+^ T cells

**Fig 4:**
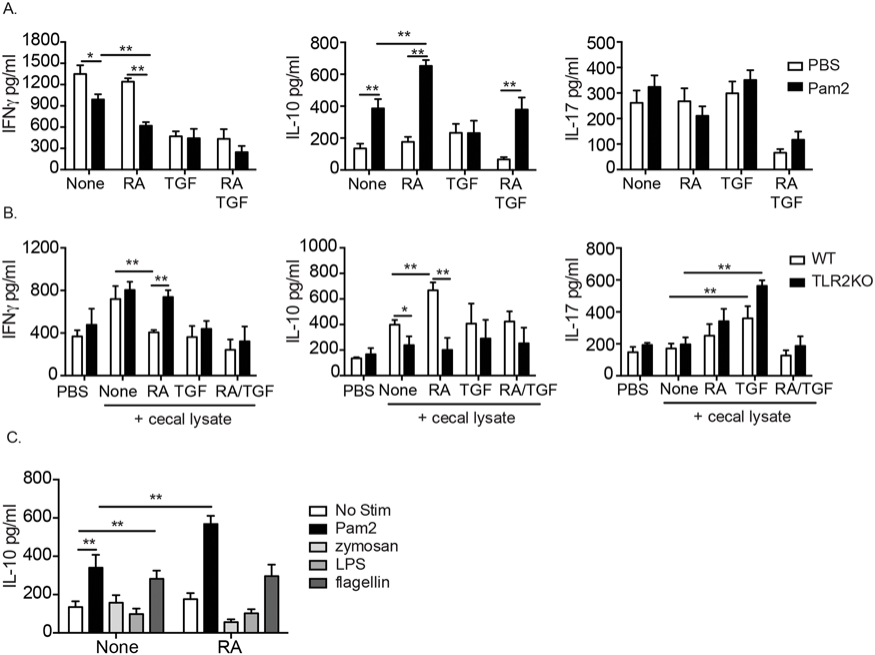
RA and TLR2 signals suppress pro-inflammatory cytokines from T cells. Cytokine production from purified splenic CD4^+^ T cells stimulated with anti-CD3ε and (A) Pam2CysK4, (B) cecal lysate, or (C) indicated TLR agonists in the presence or absence RA, TGF- or RA/TGF- β. Data are the mean ± SEM of 3–4 independent experiments. *, p < 0.05, **, p < 0.01 using Student’s t-test.

Direct signaling via TLR2 on iT_REG_ has been shown to affect their expansion and function [40, 41] and the role of RA in iT_REG_ function has been clearly established [21,23,45]. We questioned whether the ability of RA to promote iT_REG_ responses could be influenced by TLR2 signaling, which may explain the marked difference in response to RA we observed *in vivo*. To address this question, naïve splenic CD4^+^ T cells were stimulated polyclonally in the presence of Pam2. Engagement of TLR2 by Pam2 directly on T cells suppressed IFN-γ and increased IL-10 production in WT T cells. Interestingly, RA exacerbated both the IFN-γ suppression and IL-10 production (Fig 4A) suggesting an adjuvant effect of RA on TLR2-mediated T cell cytokine production. There was no effect by either TLR2 activation or RA treatment on IL-17 production (Fig 4A).

To determine whether RA could alter T cell responses in a TLR2-dependent manner in the presence of endogenous microbial ligands, we stimulated WT and TLR2KO CD4 T cells in the presence of cecal lysate, TGF- β as well as RA. Unlike the pure TLR2 agonist, Pam2, cecal lysate increased IFN-γ production by CD4 T cells, which is likely the result of the presence of alternative TLR-ligands in the culture (Fig 4B). Importantly, however, RA dramatically suppressed IFN-γ production by CD4 T cells in the presence of cecal lysate and this was not observed in TLR2KO cells (Fig 4B). Cecal lysate also increased IL-10 production by WT CD4 T cells and similar to the Pam2 stimulated cells, RA enhanced this phenotype in a TLR2-dependent manner (Fig 4B). IL-17 production was increased by cecal lysate in the presence of exogenous TGF- β, but this was not altered by either RA treatment or TLR2 deficiency. Because of the abundance of potential TLR ligands in the cecal lysate and because a recent study has shown a relationship between yeast zymosan, TLR2/dectin-1 signaling and IL-10 in DC [49], we checked to see if other TLR ligands induced IL-10 from T cells and if there was an adjuvant effect mediated by RA. The TLR5 ligand, flagellin, was the only TLR agonist screened that induced IL-10 from T cells, however RA had no effect on IL-10 levels (Fig 4C). Zymosan, which is a Dectin-1 and TLR2 agonist, was unable to induce IL-10 from purified, polyclonally stimulated T cells (Fig 4C). These data illustrate that zymosan cannot induce IL-10 from polyclonally stimulated T cells *in vitro*, and has no role in RA-mediated immune-regulation. Taken together, we conclude that in the presence of specific TLR2 ligands, RA suppresses IFN-γ and enhances IL-10 production by CD4 T cells and this is dependent on CD4 T cell intrinsic TLR2 signaling.

### RA and TLR2 synergize to promote the generation of Foxp3^+^ T cells in an IL-10, T cell intrinsic manner

**Fig 5:**
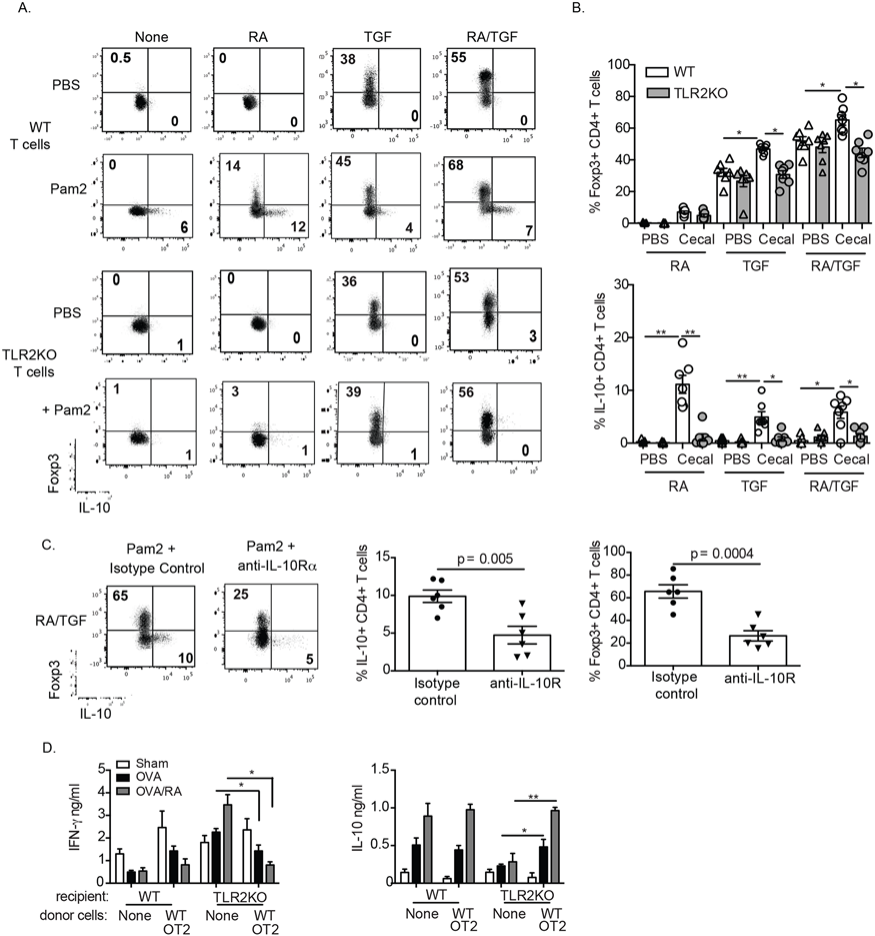
TLR2 and RA act in concert to potentiate iT_REG_ generation via IL-10 production. Foxp3 and IL-10 expression by WT and TLR2KO T cells stimulated with Pam2CysK4 (A) or cecal lysate (B) in the presence or absence RA, TGF-β or RA/TGF-β. Data are representative plots from three independent experiments for (A) and mean and frequency of Foxp3^+^IL-10^+^ and IL-10^+^CD4^+^ T cells from WT (white bars) and TLR2KO (gray bars) treated with cecal lysate (n = 5–7 mice) for (B). (C) Frequency of Foxp3 and IL-10 expression in WT T cells cultured with Pam2Cysk4, RA and TGF-β and treated with anti-IL-10Rα antibody or isotype control. Data are representative FACS plots from six independent experiments shown in the bar graphs. (D) Cytokine production from re-stimulated *in vitro* MLN cells of WT and TLR2KO mice fed OVA. Data are the mean ± SEM of two independent experiments with three mice per group. *, p < 0.05, **, p < 0.01.

To investigate the synergy of TLR2 and RA in IL-10 production by CD4 T cells, we analyzed intracellular expression of IL-10 and Foxp3 in polyclonally stimulated WT and TLR2KO CD4^+^ T cells treated with Pam2, RA and/or TGF-β. In the presence of Pam2, RA treatment caused a robust increase in IL-10^+^ CD4 T cells, which were Foxp3^-^ and this was not observed in the absence of Pam2 or in TLR2KO CD4 T cells (Fig 5A). Furthermore, in the presence of only Pam2, RA treatment caused a robust increase in non-IL-10 producing Foxp3^+^ CD4^+^ T cells and this same Pam2-mediated increase was also observed after Foxp3^+^ CD4^+^ T cells were enhanced with exogenous TGF-β (Fig 5A). Importantly, the effect of Pam2 and RA to increase Foxp3^+^ CD4^+^ T cells was not observed when TLR2KO cells were used (Fig 5A, bottom panels). Similar data was also obtained with T cells stimulated with cecal lysates instead of Pam2 alone and summarized in Fig 5B.

We hypothesized that the synergy of TLR2 and RA in increasing IL-10 production was itself driving subsequent Foxp3^+^ and IL-10^+^ CD4 T cell expansion in these cultures. To test this we added IL-10Rα blocking antibodies to CD4^+^ T cell cultures stimulated with Pam2, TGF-β and RA. Anti-IL-10Rα blockade significantly ablated both IL-10^+^ and Foxp3^+^ CD4^+^ T cell production, showing that the effect of Pam2 and RA to promote regulatory T cell responses is IL-10 dependent (Fig 5C).

### Endogenous TLR2 signals promote oral tolerance via T cells

The importance of RA in the induction of oral tolerance via generation of iT_REG_ is well established [21–23]. However, the function of endogenous microbial signals in the context of food-derived metabolic signals in this process is not well understood. Based on the above observation that TLR2 signaling on CD4 T cells synergizes with RA to promote the generation of iT_REG_ *in vitro*, we hypothesized that the absence of TLR2 may impair tolerance against orally administered proteins. To test whether RA and TLR2 work in vivo to enhance iT_REG_ numbers, WT or TLR2KO mice were fed OVA with or without concomitant RA supplementation. Two days after the last feeding, mesenteric lymph node (MLN) cells were re-stimulated *in vitro* to assess antigen-specific immune polarization. As expected, RA enhanced the normal tolerogenic response to fed OVA in WT mice by significantly increasing IL-10 and suppressing IFN-γ and IL-2 (Fig 5D, data not shown) in a TLR2 dependent manner. This was further demonstrated by the elevated pro-inflammatory IFN-γ from TLR2KO and the inability of these mice to produce IL-10 (Fig 5D). To asses whether the phenotype observed is due to intrinsic TLR2 signaling on T cells we transferred naïve OVA-specific CD4 OT2 T cells from WT mice to WT and TLR2KO mice. When these mice were fed OVA to induce tolerance, we found a reduction in IFN-γ and an induction of IL-10, similar to WT tolerized controls (Fig 5D). These data confirm that TLR2 signaling on T cells is required for oral tolerance and enhancement thereof by RA.

## Discussion

Numerous studies have delineated the role of RA in lymphocyte trafficking and differentiation [10,14,16,46]. However, the innate signaling pathways that modulate these effects are not well understood. There is evidence that RA promotes divergent immune responses depending on the environmental milieu [24,33] and that TLR2 is involved in propagating these responses [30]. Our findings illustrate a novel relationship between dietary metabolites and specific signals from the microbiome to generate tolerogenic responses. Importantly, we have shown that RA is capable of inhibiting colonic disease and this is dependent upon TLR2 as seen by decreased pro-inflammatory cytokine production, better histology scores and less bleeding. It is noteworthy that in the absence of TLR2 signaling, RA is altered from a tolerogenic adjuvant to a promoter of hyper-inflammation, drawing a close tie between RA and TLR2 signaling. In the context of DSS-induced tissue injury, our data illustrate a protective role for TLR2 signaling as TLR2-deficient mice displayed shorter colons, more weight loss and worse histological features. The addition of RA during DSS had a protective anti-inflammatory response associated with an increase in Foxp3^+^ and Foxp3^+^ IL-10^+^ expressing cells in WT mice. The effects of RA were lost in the TLR2KO mice and in fact worsened disease. These data lead us to propose a model in which IL-10 is produced during DSS-induced injury via commensal activation of TLR2 on mucosal T cells. The addition of RA increases IL-10 production by TLR2-stimulated T cells, which favors an environment that promotes the generation of Foxp3^+^ Treg cells. These data align with our previous work that suggests RA can act as an adjuvant to promote dominant responses in the mucosal tissue [33]. However, It is possible that RA may promote TLR2-induced IL-10 through another one of its signaling mechanisms.

These data illustrate that RA and TLR2 signal directly via the T cell to increase the production of IL-10. Further, we demonstrate that the IL-10 produced by RA and TLR2 increases the generation of Foxp3^+^ T cells. TLR2 has been shown to be expressed and impact the function of differentiated, activated T_REG_ [40, 41]. In fact, human T cells have been shown to induce TLR2 upon activation and Pam3Cysk4 acts as a co-stimulatory molecule inducing IL-10, TNF-α, IL-2 and IFN-γ production [47]. Our data demonstrates an important role for RA in TLR2-mediated *de novo* generation of Foxp3^+^ iT_REG_ from naïve T cells independent of TGF-β. However, when TGF-β is added to the cultures the numbers of Foxp3^+^ T cells are increased even further by the addition of TLR2 agonists. These data lead us to hypothesize that the absence of TLR2 signaling would negatively affect oral tolerance, as it appears to result in reduced numbers of iT_REG_ against endogenous or commensal antigens. We found that TLR2-deficient mice have a defect in the ability to be tolerized to oral antigen and RA administration promotes production of inflammatory responses in these mice. However, tolerance was restored in TLR2KO animals if they were given a population of TLR2-expressing, antigen-specific, naïve T cells prior to oral antigen and RA treatment.

In summary, our studies reveal that RA and TLR2 signaling synergize to promote tolerogenic responses in a T cell-dependent manner to promote tolerogenic responses via the production of IL-10 and ultimately the potentiation of iT_REG_. This not only has implications during homeostasis for the generation of tolerogenic responses against dietary antigens, but also during inflammation as a way to temper the inflammatory responses and potentially reduce damage to the tissue. Our observations indicate that enteral vitamin A supplementation or retinoid therapy at the time of diagnosis or during disease relapse may be detrimental to patients with IBD. Our findings also suggest that people with aberrant TLR2 signaling, which has been associated with the single nucleotide polymorphism R753Q, may be predisposed to inflammatory or autoimmune conditions due to impaired induction of iT_REG_, particularly in the setting of VAD [44]. Finally, given the potential of RA to transition from an essential to pathological mediator of immune responses, further investigation of how vitamin A metabolism affects disease is warranted and the molecular mechanism of TLR2 and RA interactions must be assessed.
